# Text-Book of Ophthalmology

**Published:** 1894-06

**Authors:** 


### Reviews of Books.

*Text-book of Ophthalmology.* By Dr. Ernest Fuchs. Authorised Translation from the Second German Edition. By A. Duane, M.D. Pp. xiii., 788. London: H. K. Lewis. 1893.

This volume enables all who cannot read German to become acquainted with the teaching of one of the great leaders in ophthalmology, and to study an accurate and conscientious translation of one of the best books that has ever been written on this department of surgery. Dr. Duane has added a few footnotes to amplify the text, and an appendix with illustrations of instruments commonly used in the various operations on the eye.

The subject is divided into four parts: I. Examination of the Eye, objective and functional. II. Diseases of the Eye. III. Anomalies of Refraction and Accommodation. IV. Operations. We believe this book was originally compiled as a textbook to Dr. Fuchs's lectures and demonstrations, and to prevent the necessity of note-taking in class. Four-fifths of the book is devoted to Diseases.

Conjunctivitis trachomatosa is admirably and very fully dealt with; the following conclusions are arrived at: "There is but one kind of trachoma, which, however, appears under various forms. The ultimate origin of the disease is probably

[1] *Tr. Roy. Acad. M. Ireland*, vol. xi., 1893, p. 360.

referable to the secretion of genitals affected with gonorrhœa; this secretion produces in the human conjunctiva acute blenorrhœa, which passes into chronic blenorrhœa. The secretion of the latter produces in a healthy eye directly a chronic inflammation, trachoma, which then by a repeated process of transfer spreads of itself." "Trachoma can never arise without infection." "Trachoma is very seldom met with in children. Follicular conjunctivitis is found chiefly in young people." Follicular catarrh can arise without any infection whatever; *e.g.*, atropine instillation. In histological structure no thoroughgoing distinction can be found between follicles and trachomatous granulations, but the course of the two diseases is distinct. Follicular catarrh is only slightly associated with papillary hypertrophy of the conjunctiva; it never leads to shrinking, to pannus or other sequelæ; it is a disease perfectly devoid of danger, one which even without any treatment finally gets well and leaves no trace behind. Dr. Fuchs treats trachoma by nitrate of silver solution during the inflammatory stage, and by blue-stone stick when it becomes chronic. He has no confidence in excision or expression of the outgrowth or in the cautery, or in removal of folds of conjunctiva. The sections on "Vernal Catarrh" and on "Conjunctivitis Exanthematica" are interesting. Our experience of eye affection in relation with acne or eczema has been that the cornea is usually implicated as well as the conjunctiva. Dr. Fuchs lays no stress on this point.

The chapter on diseases of the cornea is equal to the recognised difficulties of the subject. Keratitis is subdivided into suppurative and non-suppurative. The two categories are distinct, not only in their consequences, but also in their clinical aspect. Dr. Fuchs speaks highly of the curative action of the cautery in corneal ulcers, but has little confidence in the use of eserine as opposed to atropine. The prognosis of a "Foreign body in the eye" is carefully considered. Of course we should all agree that, as a rule, the eyeball requires excision; but there are exceptional cases where this need not be done if the patient is very averse to losing an eye which to external appearance is not damaged. Fuchs says, whether a body which penetrates into the eye is well borne by it or not depends upon the following circumstances: (1) Whether it is *aseptic* or not. (2) Its chemical character. Metals which, like lead and the noble metals, probably do not become oxidized in the tissues of the eye, are comparatively harmless compared with copper or iron. (3) Its weight and volume. (4) The tissues of the eye vary in respect to their tolerance of foreign bodies. Under "Iridocyclitis Sympathica," however, he says: "Most emphatically requiring enucleation are eyes which are suspected to contain a foreign body."

A good description of the anatomy of the uvea, its blood-vessels and lymph-passages; of the nourishment of the eye, and intraocular pressure; the development of the eye; and the participation of the uvea in the visual act, precedes the chapters on "Diseases of the Iris and Ciliary Body, and of the Choroid." While giving full credit to Deutschmann's experiments upon the method of transmission of sympathetic ophthalmitis, Fuchs concludes: "Whether the inflammation produced in this way is identical with sympathetic inflammation in man has not yet been certainly established. We shall have to wait for further proof before we can accept as perfectly certain the theory of transmission of the inflammation by way of the optic nerves."

Throughout the book reference is very rarely made to any English author. The chapter on "Glaucoma" is good, but would be better if a more detailed notice had been given to the careful investigations and scientific deductions of Priestley Smith in support of Knies and Weber's theory of obstruction at the filtration angle.

One of the best chapters is on "Disturbances of Motility of the Eye." It is clearly and fully written, and profusely illustrated by good diagrams. The tests for muscular insufficiency have been improved since these chapters were written; but on all matters relating to the ocular muscles, Fuchs is a well-recognised authority.

Under "Myopia" we find the generally accepted views regarding the causation of the disease. "Nearsight is an attribute of culture." "In the country we encounter fewer people with glasses than we do in the city." "The schools are the main hotbeds for the propagation of nearsightedness." "The school most dangerous for eyes is the high school." "Continuous employment of the eyes upon fine work exerts the same influence as do schools."

Dr. Fuchs insists that old people should not be confined to bed, and that in them the better eye should be left uncovered. He is an advocate of exenteration of the eyeball or even of wearing an artificial eye over the shrunken eyeball rather than its enucleation for cosmetic reasons; but he admits that irritation of the stump in many cases results. He is in favour of a small iridectomy in the extraction of cataract, and has a severe dread of any tendency to a prolapsed iris under any conditions.

In conclusion, no book can be better recommended either for the student or for the practitioner of medicine.

